# Correction: Satisfaction of scientists during the COVID-19 pandemic lockdown

**DOI:** 10.1057/s41599-020-00675-9

**Published:** 2020-12-04

**Authors:** Isabel J. Raabe, Alexander Ehlert, David Johann, Heiko Rauhut

**Affiliations:** 1 Institute of Sociology, University of Zurich, 8050 Zurich, Switzerland; 2 ETH Zurich, 8092 Zurich, Switzerland

**Keywords:** Sociology, Social policy

Correction to: *Humanities and Social Sciences Communications* 10.1057/s41599-020-00618-4, published online 4 November 2020.

Table 2 in the original paper has been corrected to ensure that the columns in the top section are aligned appropriately with those in the lower section.

In addition, the following sentence in the legend to Fig. 1 has been corrected as follows to improve clarity:

Original: For illustration purposes, this distribution was rescaled to rescaled to cover only a fraction of the figure.

Changed to: For illustration purposes, this distribution was rescaled to cover only a fraction of the figure.

